# Supplementary material for: Longitudinal evidence of technology-enhanced, individualized neuromotor rehabilitation on autonomy, cognition, quality of life, and psychological well-being: Pilot multi-sample study
Source: PLoS One. 2026 Mar 13;21(3):e0344472. doi: 10.1371/journal.pone.0344472 (PMC12987491; doi:10.1371/journal.pone.0344472)
Supplement: S1 Appendix — (PDF) [file pone.0344472.s001.pdf]

1. Stroke

1.1. Personalized technology-based intervention characteristics

Table S1. Means, standard deviations and range values for each technological device's number of training sessions and total of minute of use.

Stroke (n=11)	Armeo	Lokomat	ProKin	Walker View	D-Wall
N (%)	2(18.2)	-	6(54.5)	3(27.3)	2(18.2)
Number of sessions	11.0±1.4 (10-12)	-	12.8±6.9 (5-23)	18.0±5.3 (14-24)	5.5±0.7 (5-6)
Total minutes of use	275.0±35.4 (250-300)	-	258.3±131.1 (50-450)	386.7±110.6 (270-490)	145.0±35.4 (120-170)

1.2. Pre-post intervention analyses

Table S2. Mean scores and pre-post within- and between-group effects on functional, cognitive, HRQoL and psychological outcomes in stroke patients (n=21)

	Group 1			Group 2				ANOVA					
	Pre-	Post-	Δ	Pre-	Post-	Δ	$p_{(\Delta)}$	Time			Interaction		
								F	*p*	η^2_p	*F*	*p*	η^2_p
**Functional**													
MBI	52.3(26.5)*	62.1(30.1)°	9.8(34.5)	76.2(24.9)*	85.2(19.1)°	**9.0(11.3)**	.468	2.941	.103	.134	.005	.943	.000
MFS	35.0(13.1)	34.0(15.4)	-1.0(7.4)	28.2(19.1)	24.5(12.3)	-5.0(8.8)	.353	2.723	.116	.131	1.210	.286	.063
FIM													
Motor	45.2(15.5)*	62.3(11.9)°	**17.1(17.6)**	62.6(14.0)*	76.8(5.7)°	**14.1(9.2)**	.897	20.442	**<.001**	.561	.186	.672	.011
Cognitive	29.4(4.9)	30.5(4.8)	1.1(3.1)	31.0(3.9)	31.5(4.1)	0.5(1.3)	.897	1.804	.198	.101	.254	.621	.016
Total	74.6(18.9)*	92.8(15.4)°	**18.2(20.5)**	93.8(16.9)*	108.3(8.6)°	**14.5(8.8)**	.696	17.652	**<.001**	.525	.226	.641	.014
**Cognitive**[1]													
MoCA	23.3(3.3)	25.1(4.2)	1.8(3.1)	22.7(3.8)	25.0(2.8)	**2.4(2.9)**	.512	9.556	**.006**	.335	.198	.661	.010
SDMT	35.9(11.3)	41.9(10.9)	**6.0(6.3)**	31.8(10.6)	35.8(8.6)	**4.0(4.6)**	.557	17.365	**<.001**	.478	.719	.407	.036
TMT-A	60.9(74.9)	55.5(77.2)	-5.5(14.8)	63.4(31.4)	56.1(24.4)	-7.3(25.1)	.863	1.964	.177	.094	.042	.840	.002
TMT-B	131.3(46.6)*	119.1(45.2)°	-12.2(42.9)	298.1(205.6)*	220.9(101.6)°	**-76.4(114.4)**	.211	4.764	**.043**	.219	2.508	.132	.129
Stroop Errors	1.9(1.9)	0.5(1.1)	**-1.5(1.7)**	2.9(4.5)	2.0(2.5)	-0.9(3.2)	.282	2.096	.057	.177	.283	.601	.015
Stroop Time	19.7(9.7)	15.4(8.1)°	-4.3(7.9)	26.9(11.8)	26.6(10.5)°	-0.4(10.9)	.349	1.253	.277	.062	.884	.359	.044
FAB	14.0(4.0)	15.9(2.0)	1.9(3.5)	14.5(2.4)	16.1(1.6)	**1.7(1.9)**	.809	8.583	**.009**	.311	.037	.849	.002
Verbal fluency	33.6(9.4)	32.8(9.2)	-0.8(5.2)	30.6(11.8)	32.9(10.1)	2.4(4.3)	.132	.566	.461	.029	2.316	.145	.109
**HRQoL and Psychological**													
EQ-VAS	51.4(25.3)	76.9(19.9)	**25.5(35.4)**	43.0(22.3)	65.9(29.8)	**22.9(23.1)**	.863	14.062	**.001**	.425	.040	.843	.002
SF-12													
PCS	30.1(10.1)	37.8(6.3)	**7.7(11.9)**	34.1(6.3)	39.0(8.9)	4.9(8.3)	.557	8.167	**.010**	.301	.372	.549	.019
MCS	36.6(13.9)	50.9(9.6)	**14.2(11.6)**	37.0(11.7)	48.9(11.9)	**11.9(11.1)**	.605	27.207	**<.001**	.593	.231	.636	.012
GAD-7	9.8(7.3)	5.4(4.6)	**-4.4(4.8)**	12.4(4.8)	7.5(5.8)	**-4.9(5.8)**	1.00	15.816	**<.001**	.454	.047	.830	.002
PHQ-9	9.5(8.1)	4.9(6.2)	-4.6(6.8)	8.6(7.4)	4.5(4.5)	**-4.1(5.4)**	1.00	10.568	**.004**	.357	.036	.851	.002
SAT-P													
Mood	52.4(26.4)	60.3(23.1)	7.9(29.3)	42.5(27.6)	77.1(27.6)	**34.6(14.7)**	**.013**	18.271	**<.001**	.490	7.219	**.015**	.275
RPF	29.20(20.8)*	67.0(21.1)°	**37.8(33.0)**	58.8(33.2)*	87.9(12.5)°	**29.1(31.6)**	.426	22.476	**<.001**	.542	.381	.544	.020
ME	63.7(23.4)	71.1(16.8)	7.4(23.6)	54.0(36.1)	67.6(26.4)	13.5(28.3)	.349	3.353	.083	.150	.289	.597	.015

Values are means and standard deviations (SD). F, ANOVA Fisher's coefficient; η^2_p, partial eta squared coefficient. * Significant between-group mean differences at baseline. ° Significant between-group mean differences at post-intervention. [1] scores are adjusted for age and education. Δ Delta is the within-group difference between pre- and post-intervention mean values. Values in bold are statistically significant (Wilcoxon signed-rank test).
$p_{(\Delta)}$ is the significance from the between-group delta mean differences (Mann-Whitney test).

1.3. 6-month follow-up analyses

Table S3. Functional status at 6-month follow-up in the two study groups.

	**Group 1**	**Group 2**	**p**
BADL	5.1±1.2	5.6±0.7	.492
IADL	2.9±1.3	4.3±1.5	.062

Table S4. Baseline, post-intervention, and 6-month follow-up comparisons on EQ-VAS mean scores.

	Model assumption							
EQ-VAS	Mauchly's W	p	Sum of Squares	df	*F*	*p*	η^2_p	Power
	.958	.709						
Time			5018.8	2	5.495	**.009**	.244	.82
*Time*Group*			59.1	2	5.495	.938	.004	.06

	Baseline	Post-	Follow-up	Δ
Group 1	53.6(25.5)	76.8(21.6)	60.0(26.2)	-6.4(33.0)*
Group 2	43.0(22.3)	65.9(29.8)	53.6(19.1)	-10.6(33.3)
P	.605	.426	.442	.717

Bonferroni test was applied as post-hoc procedure
Δ Delta is the difference between baseline and follow-up mean scores
* Within-group differences are significant

Table S5. Baseline, post-intervention, and 6-month follow-up comparisons on SF-12 PCS mean scores.

	Model assumption							
SF-12 PCS	Mauchly's W	p	Sum of Squares	df	*F*	*p*	η^2_p	Power
	.971	.789						
Time			649.9	2	5.014	**.012**	.228	.78
*Time*Group*			39.4	2	.304	.740	.018	.10

	Baseline	Post-	Follow-up	Δ
Group 1	28.9(10.8)	36.4(6.1)	36.1(5.1)	-7.1(12.6)
Group 2	34.1(6.3)	39.0(8.9)	42.8(10.6)	-8.8(11.8)
p	.197	1.00	.177	.968

Bonferroni test was applied as post-hoc procedure
Δ Delta is the difference between baseline and follow-up mean scores

Table S6. Baseline, post-intervention, and 6-month follow-up comparisons on SF-12 MCS mean scores.

	Model assumption							
SF-12 MCS	Mauchly's W	p	Sum of Squares	df	*F*	*p*	η^2_p	Power
	.916	.495						
Time			1533.5	2	14.168	**<.001**	.455	.99
*Time*Group*			39.6	2	.366	.696	.021	.10

	Baseline	Post-	Follow-up	Δ
Group 1	39.3(14.1)	53.2(9.4)	48.5(14.6)	-9.2(8.3)*
Group 2	37.0(11.7)	48.9(11.9)	42.1(13.1)	-5.0(11.5)
p	1.00	.973	.238	.395

Bonferroni test was applied as post-hoc procedure
Δ Delta is the difference between baseline and follow-up mean scores
* Within-group differences are significant

Table S7. Baseline, post-intervention, and 6-month follow-up comparisons on PHQ-4 mean scores.

	Model assumption							
PHQ-4	Mauchly's W	p	Sum of Squares	df	*F*	*p*	η^2_p	Power
	.983	.875						
Time			42.8	2	4.293	**.022**	.202	.71
*Time*Group*			0.3	2	.026	.974	.002	.10

	Baseline	Post-	Follow-up	Δ
Group 1	5.4(3.7)	3.1(2.2)	3.9(3.9)	1.5(3.0)
Group 2	5.5(2.9)	3.5(2.8)	4.3(3.9)	1.2(3.2)
p	.863	.918	.778	.717

Bonferroni test was applied as post-hoc procedure
Δ Delta is the difference between baseline and follow-up mean scores

2. Parkinson's Disease (PD)

2.1. Personalized technology-based intervention characteristics

Table S8. Means, standard deviations and range values for each technological device's number of training sessions and total of minute of use.

**PD (n=8)**	**Armeo**	**Lokomat**	**ProKin**	**Walker View**	**D-Wall**
N (%)	-	2(25.0)	2(25.0)	-	4(50.0)
Number of sessions	-	7.5±3.5 (5-10)	3.5±0.7 (3-4)	-	8.3±5.4 (3-15)
Total minutes of use	-	420.0±254.6 (240-600)	67.5±3.5 (65-70)	-	150.0±45.5 (90-200)

2.2. Pre-post intervention analyses

Table S9. Mean scores and pre-post within- and between-group effects on functional, cognitive, HRQoL and psychological outcomes in patients with PD (n=12)

	Group 1			Group 2				ANOVA					
	Pre-	Post-	Δ	Pre-	Post-	Δ	$p_{(\Delta)}$	Time			Time x Group		
								F	*p*	η^2_p	*F*	*p*	η^2_p
**Functional**													
MBI	67.8(19.9)	75.8(19.5)	8.0(10.2)	75.1(22.3)	88.5(9.6)	**13.4(19.8)**	1.00	3.992	.074	.285	.252	.626	.025
MFS	50.0(17.8)	47.5(15.5)	-2.5(5.0)	31.9(12.8)	32.5(11.3)	0.6(6.8)	.570	.236	.637	.023	.656	.437	.062
FIM													
Motor	50.0(15.8)	65.8(10.3)°	15.8(6.3)	63.9(6.1)	78.1(5.6)°	**14.3(1.6)**	.648	154.028	**<.001**	.945	.366	.560	.039
Cognitive	32.5(3.1)	32.5(3.1)	0.0(0.0)	32.3(2.6)	32.9(2.1)	0.6(0.8)	.315	2.014	.190	.183	2.014	.190	.183
Total	82.5(16.3)	98.3(11.6)	15.8(6.3)	96.1(6.4)	111.0(5.9)	**14.9(1.6)**	.412	160.629	**<.001**	.947	.137	.720	.015
**Cognitive**[1]													
MoCA	22.8(5.7)	26.3(4.2)	3.4(2.6)	25.1(3.4)	25.9(4.1)	0.8(2.2)	.073	8.536	**.015**	.461	3.322	.098	.249
SDMT	41.5(20.7)	44.5(23.2)	3.0(2.6)	39.8(10.4)	45.9(14.5)	6.1(7.4)	.497	4.120	.073	.314	.483	.505	.051
TMT-A	25.4(20.8)	43.5(36.3)	18.1(20.6)	35.1(15.9)	31.9(21.6)	-3.1(16.4)	.133	1.637	.233	.154	3.242	.105	.265
TMT-B	183.2(118.9)	195.6(170.3)	12.3(52.79	171.9(131.3)	145.3(116.6)	-26.6(64.1)	.376	.117	.740	.013	.868	.376	.088
Stroop Errors	0.3(0.5)	0.6(1.1)	0.3(0.6)	2.5(4.5)	1.4(2.5)	-1.1(3.7)	.497	.128	.729	.014	.435	.526	.046
Stroop Time	38.5(29.8)	33.4(17.5)	-5.1(22.1)	20.7(21.5)	16.7(13.1)	-3.9(11.6)	1.00	.835	.358	.085	.014	.907	.002
FAB	16.1(1.7)	15.9(0.7)	-0.2(1.2)	15.5(2.1)	16.5(2.6)	0.9(1.4)	.194	.662	.437	.069	1.293	.285	.126
Verbal fluency	36.7(10.1)	36.0(10.8)	-0.7(1.5)	36.2(12.7)	38.2(12.6)	2.0(9.5)	.376	.055	.820	.006	.221	.650	.024
**HRQoL and Psychological**													
EQ-VAS	37.5(33.0)	37.5(25.0)°	0.0(21.6)	48.1(18.9)	66.3(19.8)°	**18.1(20.2)**	.214	2.063	.181	.171	2.063	.181	.171
SF-12													
PCS	25.9(10.4)	31.2(7.5)	5.3(15.1)	31.5(8.3)	37.9(10.1)	5.5(9.8)	.368	3.008	.114	.231	.028	.870	.003
MCS	39.2(15.1)	36.5(13.8)	-2.7(12.4)	40.7(15.6)	46.2(11.4)	6.4(8.7)	.808	.182	.679	.018	1.578	.238	.136
GAD-7	13.3(6.2)	11.0(5.9)	-2.3(1.3)	8.0(5.1)	5.0(4.8)	-4.4(4.3)	.570	5.799	**.037**	.367	.118	.738	.012
PHQ-9	14.3(4.7)	12.0(4.9)°	-2.3(2.5)	8.4(5.9)	4.0(3.1)°	**-3.0(4.2)**	.570	7.982	**.018**	.444	.821	.386	.076
SAT-P													
Mood	58.5(18.4)	51.5(34.6)	-7.0(38.9)	40.8(26.4)	71.6(24.7)	**30.9(25.9)**	.109	1.639	.229	.141	4.124	.070	.292
RPF	28.5(33.6)	54.8(37.1)	26.3(55.3)	45.9(34.4)	73.6(28.6)	**27.8(34.0)**	1.00	4.501	.060	.310	.003	.954	.000
ME	44.3(36.5)	65.8(38.4)	21.5(20.6)	56.3(28.5)	71.8(1.9)	**15.5(17.1)**	.808	11.033	**.008**	.525	.290	.602	.028

Values are means and standard deviations (SD). F, ANOVA Fisher's coefficient; η^2_p, partial eta squared coefficient. ° Significant between-group mean differences at post-intervention. [1] raw scores are adjusted for age and education
Δ Delta is the within-group difference between pre- and post-intervention mean values. Values in bold are statistically significant (Wilcoxon signed-rank test). $p_{(\Delta)}$ is the significance from the between-group delta mean differences (Mann-Whitney test).

2.3. 6-month follow-up analyses

Table S10. Functional status at 6-month follow-up in the two study groups.

	**Group 1**	**Group 2**	**p**
BADL	4.7±0.6	5.8±0.5	**.048**
IADL	2.0±1.0	3.3±1.3	.194

Table S11. Baseline, post-intervention, and 6-month follow-up comparisons on EQ-VAS mean scores.

	Model assumption							
EQ-VAS	Mauchly's W	p	Sum of Squares	df	*F*	*p*	η^2_p	Power
	.957	.838						
Time			875.4	2	2.080	.154	.188	.37
*Time*Group*			663.3	2	1.576	.234	.149	.29

	Baseline	Post-	Follow-up	Δ
Group 1	50.0(26.5)	50.0(0.0)	33.3(5.8)	16.6(28.9)
Group 2	48.1(18.9)	66.3(19.8)	55.0(23.3)	-6.9(18.7)
p	.461	**.048**	.194	.279

Bonferroni test was applied as post-hoc procedure
Δ Delta is the difference between baseline and follow-up mean scores

Table S12. Baseline, post-intervention, and 6-month follow-up comparisons on SF-12 PCS mean scores.

	Model assumption							
SF-12 PCS	Mauchly's W	p	Sum of Squares	df	*F*	*p*	η^2_p	Power
	.913	.694						
Time			391.1	2	4.783	**.022**	.347	.72
*Time*Group*			43.6	2	.534	.595	.056	.12

	Baseline	Post-	Follow-up	Δ
Group 1	21.2(5.5)	33.5(7.4)	24.8(2.8)	-3.6(3.6)
Group 2	31.5(8.3)	37.9(10.1)	34.3(13.1)	-2.8(9.2)
p	.461	.214	.497	1.00

Bonferroni test was applied as post-hoc procedure
Δ Delta is the difference between baseline and follow-up mean scores

Table S13. Baseline, post-intervention, and 6-month follow-up comparisons on SF-12 MCS mean scores.

	Model assumption							
SF-12 MCS	Mauchly's W	p	Sum of Squares	df	*F*	*p*	η^2_p	Power
	.775	.361						
Time			17.2	2	.095	.910	.010	.06
*Time*Group*			333.7	2	1.841	.187	.170	.33

	Baseline	Post-	Follow-up	Δ
Group 1	44.2(13.9)	37.2(16.8)	36.9(17.1)	7.2(13.8)
Group 2	40.7(15.6)	46.2(11.4)	50.3(6.2)	-9.6(14.0)
p	.683	.214	.279	.133

Bonferroni test was applied as post-hoc procedure
Δ Delta is the difference between baseline and follow-up mean scores

Table S14. Baseline, post-intervention, and 6-month follow-up comparisons on PHQ-4 mean scores.

	Model assumption							
PHQ-4	Mauchly's W	p	Sum of Squares	df	*F*	*p*	η^2_p	Power
	.774	.359						
Time			27.1	2	1.712	.209	.160	.31
*Time*Group*			6.0	2	.390	.689	.040	.10

	Baseline	Post-	Follow-up	Δ
Group 1	7.3(5.0)	6.7(5.0)	6.0(3.6)	1.3(6.7)
Group 2	4.9(3.6)	2.6(2.2)	1.3(0.9)	3.6(3.9)*
p	.283	.073	**.012**	.376

Bonferroni test was applied as post-hoc procedure
Δ Delta is the difference between baseline and follow-up mean scores
* Within-group differences are significant

3. Osteoarthritis following Total Knee/Hip Arthroplasty (TKA/THA)

3.1. Personalized technology-based intervention characteristics

Table S15. Means, standard deviations and range values for each technological device's number of training sessions and total of minute of use.

**TKA/THA (n=10)**	**Armeo**	**Lokomat**	**ProKin**	**Walker View**	**D-Wall**
N (%)	-	-	10(100.0)	-	-
Number of sessions	-	-	5.3±1.6 (39)	-	-
Total minutes of use	-	-	88.0±49.8 (40-180)	-	-

3.2. Pre-post intervention analyses

Table S16. Mean scores and pre-post within- and between-group effects on functional, cognitive, HRQoL and psychological outcomes in patients following with TKA/THA (n=24)

	Group 1			Group 2			ANOVA						
	Pre-	Post-	Δ	Pre-	Post-	Δ	$p_{(\Delta)}$	Time			Time x Group		
								F	*p*	η^2_p	*F*	*p*	η^2_p
**Functional**													
MBI	74.4(19.4)	90.4(7.1)	**16.3(19.7)**	78.4(10.4)	87.4(10.9)	**9.0(8.5)**	.508	14.333	**.001**	.394	1.190	.287	.051
MFS	34.3(9.2)	32.5(7.0)	-1.8(6.7)	36.5(11.6)	32.5(7.9)	-4.0(9.7)	1.00	3.025	.096	.121	.443	.513	.020
FIM													
Motor	62.9(10.6)	79.2(3.3)	**16.3(12.1)**	64.2(9.0)	78.8(7.9)	**14.6(5.3)**	.794	50.783	**<.001**	.717	.164	.690	.008
Cognitive	33.9(2.5)	33.9(2.5)	0.0(0.0)	34.7(0.7)	34.8(0.7)	0.1(0.3)	.695	1.477	.238	.069	1.477	.238	.069
Total	96.7(10.6)	113.0(4.9)	**16.3(12.1)**	98.9(9.1)	113.6(8.2)	**14.7(5.2)**	.744	51.519	**<.001**	.720	.145	.708	.007
**Cognitive**[1]													
MoCA	25.7(2.1)	27.3(3.2)	**1.6(2.5)**	24.3(3.5)	25.3(3.0)	1.4(3.7)	.877	5.309	**.032**	.202	.032	.859	.002
SDMT	46.9(10.7)*	48.4(9.8)	1.5(3.4)	35.4(11.9)*	41.3(8.3)	**3.9(4.6)**	.267	9.936	**.005**	.332	1.872	.186	.086
TMT-A	30.7(29.6)	21.5(20.4)	**-9.2(12.9)**	34.9(11.9)	31.8(7.8)	-1.3(7.6)	.212	4.445	**.048**	.182	2.497	.130	.111
TMT-B	86.6(111.7)	103.8(82.3)	17.2(71.3)	108.7(64.1)	115.2(77.9)	12.0(43.9)	.815	1.093	.308	.052	.035	.854	.002
Stroop Errors	3.3(6.7)	0.4(0.8)	-2.8(6.7)	2.8(3.3)	1.5(3.1)	-0.9(2.7)	.714	2.291	.146	.103	.586	.453	.028
Stroop Time	18.3(9.1)	20.8(17.1)	2.5(13.2)	27.3(15.7)	19.1(9.9)	-5.9(8.7)	.127	.427	.521	.021	2.570	.125	.114
FAB	16.2(1.8)	17.0(1.5)	0.8(2.2)	14.6(3.4)	16.5(1.6)	1.8(2.9)	.557	6.059	**.023**	.224	.808	.379	.037
Verbal fluency	42.1(9.9)	41.1(13.3)	-1.1(6.5)	39.8(10.7)	40.4(10.6)	-1.2(8.2)	.781	.551	.466	.026	.003	.956	.000
**HRQoL and Psychological**													
EQ-VAS	61.08(15.7)	68.5(11.39)	7.5(12.6)	65.5(13.4)	74.0(15.2)	**8.5(12.3)**	.752	9.606	**.005**	.304	.038	.848	.002
SF-12													
PCS	32.0(5.1)	40.9(7.0)	**8.9(5.7)**	30.0(7.5)	37.2(8.5)	**7.2(8.1)**	.172	40.327	**<.001**	.647	.417	.525	.019
MCS	42.5(10.2)	41.8(12.2)°	-0.7(5.6)	49.9(11.6)	53.6(8.9)°	7.2(6.7)	.709	1.117	.302	.048	2.440	.133	.100
GAD-7	8.7(5.0)	7.4(5.2)°	-1.3(2.9)	8.0(5.8)	3.5(2.8)°	**-4.5(5.5)**	.138	11.148	**.003**	.336	3.441	.077	.135
PHQ-9	7.4(5.8)	5.6(4.8)	-1.8(4.1)	5.1(3.8)	2.5(2.5)	-2.6(4.0)	.709	6.842	**.016**	.237	.236	.632	.011
SAT-P													
Mood	51.1(24.7)	61.2(19.6)°	10.1(23.3)	66.4(21.2)	78.4(12.6)°	12.0(16.0)	.886	6.691	**.017**	.233	.051	.823	.002
RPF	41.4(28.5)	54.4(29.8)°	13.0(30.4)	60.1(29.5)	80.2(29.8)°	**20.1(24.6)**	.546	8.040	**.010**	.268	.370	.549	.017
ME	69.3(19.3)	62.6(22.8)°	-6.7(18.9)	75.7(20.0)	85.8(14.9)°	**10.1(14.9)**	**.019**	.222	.642	.010	5.484	**.029**	.200

Notes. Values are means and standard deviations (SD). F, ANOVA Fisher's coefficient; η^2_p, partial eta squared coefficient. * Significant between-group mean differences at baseline. ° Significant between-group mean differences at post-intervention.
[1] raw scores are adjusted for age and education. Δ Delta is the within-group difference between pre- and post-intervention mean values. Values in bold are statistically significant (Wilcoxon signed-rank test). $p_{(\Delta)}$ is the significance from the between-group delta mean differences (Mann-Whitney test).

3.3. 6-month follow-up analyses

Table S17. Functional status at 6-month follow-up in the two study groups.

	**Group 1**	**Group 2**	***p***
BADL	5.5±1.7	6.0±0.0	.563
IADL	6.1±1.6	6.4±1.7	.539

Table S18. Baseline, post-intervention, and 6-month follow-up comparisons on EQ-VAS mean scores.

	Model assumption							
EQ-VAS	Mauchly's W	*p*[1]	Sum of Squares	df	*F*	*p*	η^2_p	Power
	.660	.019						
Time			765.2	1.492	2.147	.145	.097	.35
*Time*Group*			211.6	1.492	.594	.511	.029	.13

[1] Greenhouse-Geisser correction was applied (ε = .746), as test of sphericity was violated (p<.05)

	Baseline	Post-	Follow-up	Δ
Group 1	60.8(16.3)	69.1(11.9)	61.1(14.3)	-0.3(24.9)
Group 2	65.5(13.4)	74.0(15.2)	73.5(13.3)	-8.0(20.0)
p	.341	.154	**.025**	.381

Bonferroni test was applied as post-hoc procedure
Δ Delta is the difference between baseline and follow-up mean scores

Table S19. Baseline, post-intervention, and 6-month follow-up comparisons on SF-12 PCS mean scores.

	Model assumption							
SF-12 PCS	Mauchly's W	*p*[1]	Sum of Squares	df	*F*	*p*	η^2_p	Power
	.677	.025						
Time			1578.4	1.512	17.535	**<.001**	.467	.99
*Time*Group*			464.4	1.512	5.160	**.015**	.205	.74

[1] Greenhouse-Geisser correction was applied (ε = .756), as test of sphericity was violated (p<.05)

	Baseline	Post-	Follow-up	Δ
Group 1	32.3(5.5)	41.1(7.2)	38.8(10.3)	-6.6(12.7)
Group 2	30.0(7.5)	37.2(8.5)	46.9(5.9)	-16.9(8.7)*
p	.546	.403	**.043**	**.036**

Bonferroni test was applied as post-hoc procedure
Δ Delta is the difference between baseline and follow-up mean scores
* Within-group differences are significant

Table S20. Baseline, post-intervention, and 6-month follow-up comparisons on SF-12 MCS mean scores.

	Model assumption							
SF-12 MCS	Mauchly's W	p	Sum of Squares	df	*F*	*p*	η^2_p	Power
	.747	.063						
Time			201.1	2	2.082	.138	.094	.40
*Time*Group*			244.6	2	2.532	.092	.112	.48

	Baseline	Post-	Follow-up	Δ
Group 1	41.7(10.4)	41.4(12.9)	48.7(10.0)	-6.9(12.0)
Group 2	49.9(11.6)	53.6(8.9)	51.5(6.0)	-1.5(8.4)
p	.122	**.013**	.539	.093

Bonferroni test was applied as post-hoc procedure
Δ Delta is the difference between baseline and follow-up mean scores

Table S21. Baseline, post-intervention, and 6-month follow-up comparisons on PHQ-4 mean scores.

	Model assumption							
PHQ-4	Mauchly's W	p	Sum of Squares	df	*F*	*p*	η^2_p	Power
	.895	.331						
Time			97.9	2	12.457	**<.001**	.372	.99
*Time*Group*			5.8	2	.732	.487	.034	.17

	Baseline	Post-	Follow-up	Δ
Group 1	5.3(3.6)	4.3(2.6)	2.9(2.5)	2.5(3.6)*
Group 2	4.4(3.1)	2.0(1.6)	1.0(0.8)	3.4(2.7)*
p	.666	**.031**	.131	.483

Bonferroni test was applied as post-hoc procedure
Δ Delta is the difference between baseline and follow-up mean scores
* Within-group differences are significant
