# Supplementary material for: Longitudinal evidence of technology-enhanced, individualized neuromotor rehabilitation on autonomy, cognition, quality of life, and psychological well-being: Pilot multi-sample study
Source: PLoS One. 2026 Mar 13;21(3):e0344472. doi: 10.1371/journal.pone.0344472 (PMC12987491; doi:10.1371/journal.pone.0344472)
Supplement: Attachment — Submitted filename: Response Letter to Reviewers.pdf [file pone.0344472.s005.pdf]

Point-by-point response Letter to Editor and Reviewer [PONE-D-24-33691]

To the Editor and Reviewer,

Thank you for your time and the opportunity to revise our manuscript entitled: "Longitudinal impact of technology-enhanced patient-centered neuromotor rehabilitation on autonomy, cognition, quality of life, and psychological well-being: preliminary multi-sample evidence".

Given that the revision process has taken several weeks, involving an editor reassignment and multiple clarification emails, we have included a summary of the key correspondence in this letter to provide context for the changes made and ensure full transparency.

In summary, before proceeding with the manuscript revision, we contacted the assigned Editor to clarify discrepancies that emerged from the initial editorial and reviewer comments. These discrepancies primarily concerned the framing of the manuscript as a 'pilot study', the appropriateness of including statistical reporting, and the need for clarification regarding outcome comparisons made within and between study subjects. In this letter, we report the key correspondence and explain how these issues were addressed and integrated into the revised manuscript.

Editor Comments:

While The article, "Longitudinal impact of technology-enhanced patient-centered neuromotor rehabilitation on autonomy, cognition, quality of life, and psychological well-being: preliminary multi-sample evidence," offers a comprehensive and valuable contribution to the growing body of research in neuromotor rehabilitation, the following limitations and areas should be improved:

1. Sample Size and Generalizability: *The study's small sample size across groups limits statistical power and external validity. While effect sizes are reported, a larger and more diverse sample would enhance the robustness of findings.*
2. Non-Randomized Design: *The non-randomized design introduces potential selection bias. Randomized controlled trials (RCTs) are needed to confirm the causal relationship between technology-enhanced interventions and observed outcomes.*
3. Heterogeneity in Interventions: *Participants received varying doses and types of technological interventions (e.g., VR versus exoskeleton). While this reflects real-world clinical practice, it complicates the attribution of effects to specific technologies.*
4. Limited Cognitive and Psychological Insights: *Cognitive outcomes, though promising, were not consistently significant. The lack of cognitive stimulation within the rehabilitation programs may have contributed to this limitation. Similarly, the psychological outcomes rely heavily on self-reported measures, which are prone to bias and may not fully capture nuanced changes.*
5. Underreporting of Statistical Models: *The article mentions using ANOVA and other statistical methods but lacks detailed reporting of model assumptions, adjustments, or corrections for multiple comparisons. This transparency is essential for reproducibility.*

Reviewer's comments:

The present article describes a prospective two-arm, non-randomized pilot study to obtain multiple pieces of evidence on a multi-domain impact of technology-enhanced neuromotor rehabilitation from a convenience-based sample.

Said that the paper routinely presented p-values in all the comparisons. For a pilot study which is not a powered trial the meaning and presentation of such p-values does not make much sense. As such, the purpose of conducting a pilot study is to examine the feasibility of an approach that is intended to be used in a larger-scale study. A pilot study is not a hypothesis-testing study. Therefore, no inferential statistical tests should be proposed in a pilot study protocol, and any such tests conducted in a post-hoc fashion hold little practical meaning. Without inferential statistical tests, a pilot study will not provide p-values. Power analyses are used to determine the sample size needed to achieve adequate statistical power (typically 80% or 90%) for detecting a clinically meaningful difference with the specified inferential statistical test. However, power analyses should not be included in an application for a pilot study that does not propose inferential tests.

I recommend rewriting the paper to focus on it as a 'pilot study' aimed at evaluating the feasibility of recruitment, randomization, retention, assessment procedures, new methods, and the implementation of the novel intervention. As a side note, since this is not a randomized trial, it is unclear how subjects are allocated to the two arms with nearly equal sample sizes. This process should be clearly described and further elucidated to understand its potential impact on a future randomized trial.

Correspondence (highlights):

From: cira.fundaro@icsmaugeri.it
Sent: 2/4/2025
To: plosone@plos.org

Object: Clarification on Revision Procedures Following Reviewer and Editor Comments

Dear Editor,

We appreciate your time in handling our manuscript, "Longitudinal impact of technology-enhanced patient-centered neuromotor rehabilitation on autonomy, cognition, quality of life, and psychological well-being: preliminary multi-sample evidence."

After reviewing the comments from both the previous editor and the reviewer, we noticed some discrepancies that we would like to clarify before proceeding with our revisions. Specifically, while we acknowledge and accept the reviewer's suggestion to frame the study as a 'pilot study', we do not share the perspective that statistical results should be omitted entirely. The previous editor also emphasized the importance of transparent statistical reporting, which seems to contrast with the reviewer's recommendation to remove inferential statistical tests.

Could you please provide guidance on how we should reconcile these differing perspectives in our revision?

We appreciate your clarification and look forward to your advice.

Best regards,

Cira Fundarò

From: plosone@plos.org
Sent: 2/5/2025
To: cira.fundaro@icsmaugeri.it

Dear Dr. Fundarò,

Thank you for asking for more information on this comment. I have reached out to them for clarification and will be in touch when we have a response.

I appreciate your patience, and encourage you to reach out in one week if you haven't heard back by then. If you have any questions in the meantime, please feel free to ask.

Best regards,

Roxanne Jastine Baltar

Straive Editorial Assistant

From: cira.fundaro@icsmaugeri.it
Sent: 2/13/2025
To: plosone@plos.org

Dear Dr. Baltar,

As suggested, I am following up on my previous email regarding the clarification on the discrepancies between the reviewer's comments and the previous editor's guidance. I wanted to check if there are any updates on this matter.

I appreciate your time and look forward to your response.

Best regards,

Cira Fundarò

From: cira.fundaro@icsmaugeri.it
Sent: 3/3/2025
To: plosone@plos.org

Dear Editor,

I hope this email finds you well.

I am reaching out to follow up on any updates regarding the review process for our manuscript. Since today marks the deadline for the revisions, I wanted to check if there are any developments on this matter.

I would greatly appreciate any information you can provide at your earliest convenience.

Thank you for your time and consideration.

Best regards,

Cira Fundarò

From: neurorehab@yonsei.ac.kr
Sent: 3/3/2025
To: cira.fundaro@icsmaugeri.it

Dear Authors,

It seems that your email may have been directed to the wrong recipient. As an Academic Editor, my role was to review your article and provide recommendations; however, the final decision rests with the Editor-in-Chief. I encourage you to reach out to them directly regarding the revision status and final decision.

That being said, a significant concern with your study lies in its design—particularly the comparison between subjects with neurological and musculoskeletal impairments, as well as the unequal sample size. A more rigorous approach would have been to compare subjects within a more homogeneous neurological population while maintaining a balanced sample size. This issue presents a substantial limitation, and I am uncertain whether it can be effectively mitigated or revised at this stage. However, other reviewers may have differing perspectives that support your current design. I am sorry that I can not support your current study design. I appreciate your efforts and encourage you to seek guidance from the Editor-in-Chief for further clarification.

Best regards,

Joshua

From: cira.fundaro@icsmaugeri.it
Sent: 3/3/2025
To: chenem25@gmail.com

Dear Dr. Emily Chenette, Ph.D.,

I hope this email finds you well. I am reaching out to request clarification regarding the status of our manuscript submitted to PLoS ONE entitled "Longitudinal impact of technology-enhanced patient-centered neuromotor rehabilitation on autonomy, cognition, quality of life, and psychological well-being: preliminary multi-sample evidence."

We initially sought guidance on how to reconcile the discrepancies between the reviewer's comments and the previous editor's recommendations, particularly regarding the statistical reporting. However, despite our repeated follow-ups over the past weeks, we have yet to receive clear guidance and were ultimately advised to contact you directly regarding the revision status and final decision. Given that today marks the deadline for submission, we would appreciate your guidance on how to proceed with our revisions to align with the journal's expectations. Specifically, we would like to understand how to address the conflicting recommendations, ensure our revision meets the required standards, and clarify the next steps in the process.

We would be grateful for your time and any direction you can provide.

Best regards,

Cira Fundarò

From: plosone@plos.org
Sent: 3/12/2025
To: cira.fundaro@icsmaugeri.it

Dear Dr. Fundarò,

Thank you for contacting us with this request, and thank you for submitting your work to PLOS One.

We have now received comments from the Academic Editor. Their comments are as follows:

"I fully support the transparent reporting of statistical results in the paper. However, I believe the reviewer's suggestion to remove the inferential statistical results may have stemmed from the fact that the study has a significant design flaw—namely, comparing two fundamentally different conditions: neurological impairment (apples) and musculoskeletal impairment (oranges). This inappropriate comparison raises concerns about the study's validity from the outset."

Please note that you can always disagree with the comments provided by editors or reviewers, so long as you provide your reasoning for the disagreement in the Response to Reviewers file submitted alongside your manuscript.

I hope this assists you in your revision process. If you have any further questions or concerns, please do not hesitate to let me or another staff member know.

Kind regards,

Marcelle Thomas

Publications Assistant

Point-by-point responses to Editor's and Reviewer's comments and revisions made

Editor Comments:

While The article, "Longitudinal impact of technology-enhanced patient-centered neuromotor rehabilitation on autonomy, cognition, quality of life, and psychological well-being: preliminary multi-sample evidence," offers a comprehensive and valuable contribution to the growing body of research in neuromotor rehabilitation, the following limitations and areas should be improved:

1. Sample Size and Generalizability: *The study's small sample size across groups limits statistical power and external validity. While effect sizes are reported, a larger and more diverse sample would enhance the robustness of findings.*

Overall, thank you for your note of appreciation regarding our contribution and for this first comment that allows us to better clarify that our main purpose, through this preliminary study, was to provide pilot evidence across three distinct and representative clinical populations undergoing neuromotor rehabilitation, specifically on a set of treatment outcomes that goes beyond motor functioning. We acknowledge that the small sample size across the three study groups may have limited findings robustness. Accordingly, we have mentioned this issue in "limitation" paragraph as follows:

> "[…] the sample sizes of all study groups are limited. Although satisfactory effect sizes were estimated and adequate achieved statistical power was observed, the results obtained cannot be considered as generalizable. Despite promising, the multi-sample evidence described so far should be taken as pilot and cautiously."

Additionally (also in accordance with reviewer's suggestion), we have added to limitation section a literature reference regarding sample size appropriateness for pilot clinical trials, specifically conducted within motor rehabilitation settings, which supports that 6 participants are sufficient to pilot the method and the implementation of a novel intervention or approach and, subsequently, feed trial scale-up. Here we report the integration we have made:

> "Overall, some limitations should be acknowledged from this study. Firstly, the sample sizes of all study groups are limited. **According to guidelines for pilot studies, however, a convenience sample of at least six participants per group may be sufficient for investigating recovery outcomes in pilot rehabilitation trials that are at a feasibility stage [95]. […]"**

Reference added:

[95] Dobkin BH. Progressive Staging of Pilot Studies to Improve Phase III Trials for Motor Interventions. Neurorehabil Neural Repair. 2009;23(3):197-206. doi:10.1177/1545968309331863

2. Non-Randomized Design: *The non-randomized design introduces potential selection bias. Randomized controlled trials (RCTs) are needed to confirm the causal relationship between technology-enhanced interventions and observed outcomes.*
3. Heterogeneity in Interventions: *Participants received varying doses and types of technological interventions (e.g., VR versus exoskeleton). While this reflects real-world clinical practice, it complicates the attribution of effects to specific technologies.*

Thank you for these comments, which helped us to better articulate the rationale behind the intervention, reflecting routine clinical practices at the Institution where the trial was conducted. We acknowledge that varying doses and types of interventions may introduce considerable heterogeneity, making it difficult to directly attribute outcomes to specific intervention components. However, we support the value of the real-world data collected and analyzed in this study. As described in the "Intervention" section, rehabilitation treatment design and delivery were strictly individualized, based on each patient's clinical diagnosis, disease severity, and specific rehabilitation needs. This personalized and patient-centred approach led to the development of tailored rehabilitation plans, which made participant randomization and intervention standardization unfeasible in the context of the present study. As previously mentioned, the primary aim at this phase was to pilot the effectiveness of this intervention approach - which integrates the ICD and ICF frameworks - by focusing on a set of non-motor outcomes that, according to the literature, warrant greater attention and remain underexplored. The study was conducted on a convenience sample. Here, we include intervention description and allocation modalities, and the related limitations acknowledged in discussion section (integration included in bold):

> "Patients were assigned to the two study arms, depending on their rehabilitation program (Figure 1). This was defined following the routine multidisciplinary clinical practice of the Institution where the study was carried out [27]. In particular, each patient's recovery pathway was designed individually based on the integration between the International Classification of Diseases (ICD) and the International Classification of Functioning, Disability, and Health (ICF) models [28]. Therefore, based on their rehabilitation needs, patients could have participated in further activities besides physical therapy, namely occupational therapy, speech therapy, cognitive stimulation, and/or psychological support. Accordingly, rehabilitation procedures followed a biopsychosocial approach and were adapted according to one's specific diagnosis, disability severity, and recovery objectives, ultimately delivering personalized rehabilitation projects and programs."
>
> […]
>
> "Another limitation concerns the intervention characteristics. As already described, patients participated in different rehabilitation activities and experienced the use of different technological devices, at different dose, based on their individual rehabilitation project and program. Although this contributed to obtaining greater within-group heterogeneity in terms of intervention delivered, the multidisciplinary and tailored approach adopted reflects the attempt to place the present study within a personalized medicine framework, whose adoption still represents one of the major challenges in the field of rehabilitation medicine [3,28,95]. **Moreover, this also aligns with the study's purpose of exploring intervention impact on a set of non-motor outcomes that are still insufficiently studied despite their recognized relevance.** Moving forward, future research may further investigate technology widespread impact through the adoption of more controlled study designs (e.g., RCTs) in order to provide integrating evidence"

4. Limited Cognitive and Psychological Insights: *Cognitive outcomes, though promising, were not consistently significant. The lack of cognitive stimulation within the rehabilitation programs may have contributed to this limitation. Similarly, the psychological outcomes rely heavily on self-reported measures, which are prone to bias and may not fully capture nuanced changes.*

Thank you for this insightful comment. We acknowledge the limitations associated with the assessment of cognitive and psychological outcomes in the present study. As noted in the manuscript, psychological outcomes were assessed using self-report measures, which - despite their practicality and alignment with the patient-centred approach of our intervention - are indeed prone to biases (e.g., social desirability) and may not fully capture intended changes. In response to this comment, we have expanded the relevant discussion section to explicitly recognize the limitation concerning the absence of structured cognitive stimulation within the intervention, which may have contributed to the non-significant trends observed in cognitive outcomes. We have also acknowledged the potential benefit of integrating more objective assessments (e.g., performance-based or clinician-rated tools) in future studies to enhance the reliability of psychological and cognitive outcome evaluation. Integrations are reported in bold:

> "The last limitation of this study concerns the use of self-report measures to investigate HRQoL and psychological outcomes. Although self-report methods may be constrained by inferential limitations (e.g., social desirability bias, risk of false positives), they remain suitable for capturing patient experiences and are valued for their practicality, clinical relevance, and cost-effectiveness [96]. Moreover, their adoption aligns with the patient-centred approach of the present study. **Nonetheless, we acknowledge that relying exclusively on self-reported data may not fully capture individual changes. Similarly, the absence of structured cognitive stimulation within the rehabilitation programs may have limited the extent of cognitive improvements observed, which were not consistently significant across cognitive sub-domains. Future studies should consider including targeted cognitive training components and integrate more objective metrics.**"

5. Underreporting of Statistical Models: *The article mentions using ANOVA and other statistical methods but lacks detailed reporting of model assumptions, adjustments, or corrections for multiple comparisons. This transparency is essential for reproducibility.*

We appreciate this comment regarding the need for greater transparency in the reporting of statistical methods. In response, we have ensured that all statistical coefficients reported in the manuscript are accurate and correspond to the analysis outputs. Additional statistical details, specifically regarding model assumptions, corrections, and full test statistics, have been provided in the Supplementary Material on 6-month follow-up analyses where repeated measures ANOVA models including three time points were conducted. For these analyses, Mauchly's test of sphericity was performed, and adjustments (e.g., Greenhouse–Geisser) were applied where necessary. For repeated measures ANOVAs involving only two time points, the sphericity assumption was not tested, as it is automatically satisfied. Post hoc comparisons were conducted using Bonferroni correction, and these are also documented in the Supplementary Material (as an extension of the previously provided follow-up analyses at six months). We believe these additions enhance the transparency and reproducibility of our statistical approach, while keeping the main manuscript concise and focused. Here we report the integration made in "Data analysis" paragraph regarding model assumption check method adopted (in bold):

> "[…] Further models were finally tested on the outcomes evaluated at 6-month follow-up (i.e., HRQoL, anxiety and depression symptoms). **For each of them, assumption of sphericity was assessed through Mauchly's test. In cases assumption violation, appropriate adjustments were applied. For all pairwise comparisons, Bonferroni correction was used (see supplementary material for further details).** Partial eta squared ($\eta^2 p$) coefficients as indicator of effect size were generated along with achieved statistical power."

In addition, we have revised the figures related to the longitudinal (6-month follow-up) analyses to improve both readability and image quality. These updated visuals aimed to enhance the clarity of results presentation and have been uploaded as part of the re-submission materials.

<u>Reviewer's comments:</u>

The present article describes a prospective two-arm, non-randomized pilot study to obtain multiple pieces of evidence on a multi-domain impact of technology-enhanced neuromotor rehabilitation from a convenience-based sample.
Said that the paper routinely presented p-values in all the comparisons. For a pilot study which is not a powered trial the meaning and presentation of such p-values does not make much sense. As such, the purpose of conducting a pilot study is to examine the feasibility of an approach that is intended to be used in a larger-scale study. A pilot study is not a hypothesis-testing study. Therefore, no inferential statistical tests should be proposed in a pilot study protocol, and any such tests conducted in a post-hoc fashion hold little practical meaning. Without inferential statistical tests, a pilot study will not provide p-values. Power analyses are used to determine the sample size needed to achieve adequate statistical power (typically 80% or 90%) for detecting a clinically meaningful difference with the specified inferential statistical test. However, power analyses should not be included in an application for a pilot study that does not propose inferential tests.
I recommend rewriting the paper to focus on it as a 'pilot study' aimed at evaluating the feasibility of recruitment, randomization, retention, assessment procedures, new methods, and the implementation of the novel intervention. As a side note, since this is not a randomized trial, it is unclear how subjects are allocated to the two arms with nearly equal sample sizes. This process should be clearly described and further elucidated to understand its potential impact on a future randomized trial.

We thank the reviewer for this important general observation, which prompted a careful reflection on how to better communicate the exploratory and feasibility-oriented nature of our work. Following the receipt of the reviewer's comments, we reached out to the journal's editorial team to clarify the apparent discrepancy between this recommendation and prior editorial guidance, which emphasized the importance of transparent statistical reporting. As documented in the correspondence highlights included in the present response letter introduction, the journal ultimately confirmed that statistical reporting - including inferential results - is acceptable, particularly if appropriately contextualized, as was our intention.
Accordingly, we have revised the manuscript to ensure that its pilot nature is explicitly and consistently stated throughout the manuscript, while clearly acknowledging that statistical analyses were conducted via an exploratory approach, rather than confirmatory. In summary, here we highlight the revisions made throughout the manuscript:

- We have explicitly defined the study as a pilot investigation throughout the abstract, introduction, methods, and discussion, emphasizing its exploratory and hypothesis-generating nature.
- We have described inferential statistics as illustrative only and that these were intended to describe trends rather than support confirmatory conclusions.
- In discussion and limitation sections, we have underlined that statistical comparisons are preliminary, potentially underpowered, and intended to guide future research and interventions.

Moreover, we would like to clarify the apparent misunderstanding regarding the structure of the comparisons made in our analyses. As further clarified in our correspondence with the editorial team, we did not perform direct statistical comparisons between different clinical groups (i.e., stroke vs. PD vs. OA). Rather, the three groups of patients were analyzed independently from one another. Indeed, no comparisons were described in the discussion section either. Here we report the parts of the manuscript text where such statistical approach was made explicit:

> Data Analysis
> "[…] All the statistical analyses were replicated for each study sample involved (i.e., stroke, PD, and OA) and described independently."
>
> Discussion
> "[…] Pre- post-intervention changes and longitudinal effects over 6 months were estimated, and within- and between-group comparisons were made for each study sample considered independently."

We acknowledge that the quality of the second sentence reported in discussion section can be improved. Here we report a revised version to ensure clarity and readability:

> "**Pre- post-intervention changes and longitudinal effects over 6 months were analysed estimating intra- and inter-group effects. All analyses were conducted separately for each clinical sub-sample (i.e., stroke, PD, and OA), and the corresponding results were interpreted independently**."

As a final note, we appreciate reviewer's observation regarding participants allocation procedure. As already described in "Intervention" paragraph, participants were not randomized but were assigned to the two intervention arms based on routine clinical decision-making at the Institution. This process involved a multidisciplinary assessment of each patient's functional status, medical condition, and rehabilitation needs, following standard care protocols. Although this study adopted a non-randomized design, the approximate balance in group sizes occurred naturally due to the rehabilitation service's effort to evenly distribute available technological resources and ensure timely access to care for all eligible patients. Thus, the nearly equal sample sizes were not the result of any formal assignment or stratification procedure, but rather an outcome of operational logistics in a real-world clinical setting. As detailed, in both the Methods and Discussion sections, each sub-sample (Stroke, PD, and OA) was analyzed independently, with no comparisons made across groups. The study's purpose was to provide feasibility evidence from condition-specific data and a multi-domain evaluation of technology-enhanced, patient-centred rehabilitation. To further elucidate this process, we propose to add the following integration to methods section (in bold):

> "Patients were assigned to the two study arms, depending on their rehabilitation program (Figure 1). This was defined following the routine multidisciplinary clinical practice of the Institution where the study was carried out [27]. In particular, each patient's recovery pathway was designed individually based on the integration between the International Classification of Diseases (ICD) and the International Classification of Functioning, Disability, and Health (ICF) models [28]. Therefore, based on their rehabilitation needs, patients could have participated in further activities besides physical therapy, namely occupational therapy, speech therapy, cognitive stimulation, and/or psychological support. Accordingly, rehabilitation procedures followed a biopsychosocial approach and were adapted according to one's specific diagnosis, disability severity, and recovery objectives, ultimately delivering personalized rehabilitation projects and programs. **Although participants did not undergo randomization, the approximate balance in sample sizes across study arms and study groups resulted from Institution's routine effort to optimize resource allocation and ensure equitable access to care, reflecting real-world clinical practice.**"
