# Supplementary material for: Longitudinal evidence of technology-enhanced, individualized neuromotor rehabilitation on autonomy, cognition, quality of life, and psychological well-being: Pilot multi-sample study
Source: PLoS One. 2026 Mar 13;21(3):e0344472. doi: 10.1371/journal.pone.0344472 (PMC12987491; doi:10.1371/journal.pone.0344472)
Supplement: Attachment — Submitted filename: Response_Letter_to_Reviewers_auresp_2.pdf [file pone.0344472.s006.pdf]

Point-by-point response Letter to Editor and Reviewer [PONE-D-24-33691R1]

To the Editor and Reviewers,

We would like to thank you for your time and for the careful evaluation of our manuscript entitled: "Longitudinal impact of technology-enhanced patient-centered neuromotor rehabilitation on autonomy, cognition, quality of life, and psychological well-being: pilot multi-sample evidence".

We are grateful for the constructive comments received during this second round of review. We are pleased that Reviewer #3 found the revised manuscript suitable for publication, and we sincerely thank the other Reviewers for their additional comments, which further helped us to improve the clarity, rigor, and overall quality of our work.

We have carefully addressed all remaining comments and suggestions. Revisions have been incorporated into the manuscript and, below, we provide a detailed point-by-point response to each comment. We hope that the revised version satisfactorily addresses all concerns and we thank you again for the opportunity to further enhance our research.

Reviewer #2: Comments to the authors:

General comments:

All acronyms and abbreviations should be mentioned (example ADLs).

Thank you for this suggestion. We have mentioned all acronyms and abbreviation when needed and consistently throughout the manuscript text.

The inclusion of OA is not a typical neuromotor disease??? The rationale of their inclusion needs to be stated?

Thank you for this valuable comment, which further helps us to better clarify the rationale behind the inclusion of OA patients in our study. We agree that OA is not a neurological condition. Its inclusion was basically motivated by the rehabilitation context rather than by disease etiology. Specifically, patients undergoing TKA/THA typically follow structured neuromotor rehabilitation programs aimed at recover motor control, balance, gait and autonomy. Within an ICF-based and biopsychosocial framework, OA therefore represents a musculoskeletal condition allowing us to explore the feasibility and multi-domain impact of technological rehabilitation across heterogeneous but representative clinical population. We have clarified this rationale in the Introduction paragraph (changes in bold):

> "[…] For this purpose, a preliminary longitudinal investigation was conducted in convenience samples of patients drawn from three distinct but representative clinical populations typically undergoing neuromotor rehabilitation, namely stroke (neurological sub-acute), PD (chronic neurodegenerative), and OA (musculoskeletal). **The latter was included as a representative musculoskeletal condition commonly undergoing structured neuromotor rehabilitation, particularly in the post-surgical phase**"

Specific comments:

The methods section of the abstract did not mention important details about what personalized technology enhanced neuromotor rehabilitation used for each group compared to standard training??
Post intervention period for each subgroup??? Were all standardized at 4 weeks?
What do you mean by interaction effects??? Line 40.
Line 41: is not clear, evidencing wider short time effects???
How this time effect was estimated?? Line 42.

Thank you for all these precious comments and suggestions on abstract's details. Here we report the 'Methods' and 'Results' sections including the revisions made while preserving a concise abstract format (changes in bold):

> Methods
>
> A prospective, two-arm, non-randomized study design was adopted to provide pilot feasibility evidence on the multi-domain impact of personalized technology-enhanced neuromotor rehabilitation from convenience sub-samples of patients with stroke, Parkinson's Disease (PD), and osteoarthritis (OA). **Technological intervention consisted of the integrated use of robot-assisted and/or VR-based exercises, individualized based on patient's diagnosis and rehabilitation goals.** Study outcomes included patient's functional status (autonomy in ADLs, risk of falls), cognition (attention and executive functions, memory, verbal fluency), physical and mental health-related

quality of life (HRQoL), and psychological status (anxiety and depression symptoms, and well-being) and were compared to patients participating in standard individualized training only. Rehabilitation experience and technology psychosocial impact were also evaluated. Intra- and intergroup comparisons along with general linear models were statistically tested within each sub-sample considered independently over three timepoints (baseline, **4-week** post-intervention, 6-month follow-up).

Results

At post-intervention, significant multi-domain intra-group improvements were observed within each sub-sample. Between-group differences were found on ADLs autonomy (stroke and PD), executive functions (stroke), anxiety and depression (OA and PD, respectively), and well-being (stroke and OA). Interaction effects **(time x group)** were significant only on well-being variables in stroke and OA, evidencing wider short-term effects of technology-enhanced programs **compared to standard training**. At 6-month, significant time effects **over the three timepoints** were estimated on HRQoL within each sub-sample and, additionally, on anxiety and depression in stroke and OA. Interaction effects emerged only on physical HRQoL in OA, along with significant between-group differences on HRQoL and anxiety and depression in OA and PD, respectively.

Introduction:

Extensive literature on the three pathological (diseases) conditions without providing details on how enhanced technology would alleviate consequences and complications, accelerate restoration of the multi-domain functionality, and improving the patient's outcomes????
The rationale for study conduction is not fully supported by the given background or the introduction????
Previous literature on how such personalized technology-enhanced motor rehabilitation are scarce and inadequate.

Thank you for this important comment. We agree that the already revised version of the introduction placed emphasis on the clinical burden of the three conditions but lacks details on the specific mechanisms and advantages of technology-enhanced rehabilitation to support multi-domain recovery. Accordingly, we have revised the introduction to explicitly describe key benefits of robotic and VR-based training based on most recent literature on stroke, PD and OA. This revision was intended to strengthen the theoretical rationale while keeping a concise narrative and highlighting the existing gap in evidence on multi-domain rehabilitation outcomes (integration in bold):

"[…] Although the efficacy of technology to improve motor impairment has been supported widely [5-10], understanding the short- and long-term broader impact on patients' non-motor characteristics still represents an open challenge [24]. **Beyond motor recovery, emerging evidence suggests that technological devices may support multi-domain rehabilitation through several mechanisms, including the delivery of high-intensity and task-specific training, the provision of augmented multisensory feedback, and the facilitation of motor-cognitive integration. In post-stroke rehabilitation, robotic and VR-based interventions have been associated with improved motor learning and engagement, as well as potential secondary benefits on attention, executive functions, and mood [5-6]. In PD, technology-enhanced training has been shown to promote gait automatization, balance control, and dual-task performance, while also positively influencing motivation and perceived well-being [7-8]. Similarly, in OA and post-arthroplasty rehabilitation, VR-based interventions may enhance proprioception, balance, and functional mobility, while reducing risk and fear of falls and psychological distress [9-10]. Collectively, these advantages support the potential of rehabilitation technologies to restore functional limitations, mitigate secondary complications, and promote recovery across physical, cognitive, and psychological domains.** Clearer evidence in this direction not only would extend knowledge on technology effectiveness to patient's global functioning, but it would also corroborate the added value of providing comprehensive and technology-enhanced recovery programs […]"

The aim of this pilot study was to provide feasibility evidence? Please elaborate???
There is hypothesis (or hypotheses) for this longitudinal study???? Or the pilot

Thank you for these important comments which allowed us to better refine our study aims and hypotheses. By 'multi-domain feasibility evidence' we refer to the preliminary evaluation of the applicability and potential added value of individualized technology-enhanced neuromotor rehabilitation in real-world clinical settings, as well as its ability to capture changes across multiple outcome domains. Although the study was not designed to test confirmatory hypotheses, we had formulated priori exploratory expectations consistent with a pilot longitudinal design. Here we propose the following integration to better refine our research purposes (integrations in bold):

"Following this line, the present study aimed to provide **pilot multi-domain feasibility evidence** on the effectiveness of multidisciplinary, individualized, technology-enhanced neuromotor rehabilitation programs. **Feasibility was intended as the preliminary assessment of the applicability and potential added value of**

technology-integrated rehabilitation within real-world clinical settings, as well as its sensitivity in capturing changes across multiple outcome domains. For this purpose, a preliminary longitudinal investigation was conducted […]. **In line with the pilot and exploratory nature of the study, it was hypothesized that, compared to patients undergoing standard multidisciplinary rehabilitation alone, those participating also in technology-enhanced programs would show better short-term multi-domain trends and a more positive rehabilitation experience. It was further exploratorily expected that improvements in psychological well-being and HRQoL would be at least partially maintained at follow-up**."

Line 108 to line 118: this section should be part of the methods section with details and does not belong to the introduction or the background section.

Thank you for this comment. We acknowledge that this section includes methodological elements related to sampling and study outcomes. However, we preferred to retain it in the Introduction, as it was intended to support the study rationale by clarifying the choice of clinical populations and the real-world context in which the intervention was implemented. Detailed method information is fully reported in the Methods section. We believe that keeping this brief description in the Introduction helps reader better understand the conceptual framework and the objectives of the study.

Methods:

Line 129: the time elapsed between study conduction and submission is almost three years!!! Authors should provide proper justification for such time gap???

Thank you for raising this point. The time gap between study conduction and manuscript submission and revision was not due to delays in data analysis or reporting by the authors, but rather to external and organizational factors related to the clinical and institutional context where the study was carried out. Importantly, the study design, data collection procedures, and outcome measures remained consistent throughout this period, and the validity of the findings was not affected.

Again all acronyms and abbreviations should be mentioned (line THA, ----)

Thank you again for pointing this out. As already mentioned, we have mentioned all acronyms and abbreviations as appropriate throughout the manuscript text.

Line 160 to line 163: it is a non-randomized pilot trial; this section is not needed?

Thank you for this suggestion. This section was added from previous revision according to reviewer's comment, that asked for clarifications concerning patient allocation in the absence of randomization. Given the non-randomized design, we considered this brief clarification important to ensure transparency and to contextualize the balance observed across study arms as a reflection of routine clinical practice rather than an allocation strategy. Accordingly, we preferred to retain this section.

How the technology-enhanced group was assigned???

Thank you for this comment. The assignment of patients to technology-enhanced group is described in detail in the "Intervention" paragraph. Briefly, patients were allocated to study arms based on their individualized rehabilitation programs and objectives, which were defined according to the Institution's routine clinical practice. Specifically for technology-based program assignment, eligibility and its implementation followed rehabilitation technology-specific guidelines and recommendations. Here we report the extracts from the manuscript where these details are described:

"Patients were assigned to the two study arms, depending on their rehabilitation program and objectives (Figure 1). These were defined following the routine multidisciplinary clinical practice of the Institution where the study was carried out [27]. In particular, each patient's recovery pathway was designed individually based on the integration between the International Classification of Diseases (ICD) and the International Classification of Functioning, Disability, and Health (ICF) models [28]. […] Only the patients assigned to the second study group arm underwent technology-based training. For these, part of the duration (which depended on rehabilitation objectives) of the whole intervention consisted of the use of exoskeletal (Lokomat®, Armeo-Spring® – Hocoma AG, Switzerland) and/or VR technology (ProKin, D-Wall, Walker View – TecnoBody SRL, Italy), resulting in the same amount of therapy for all

> participants. Technology-enhanced rehabilitation procedures, including eligibility criteria for assignment and implementation, followed appropriate rehabilitation technology-specific guidelines and recommendations [29–33].”

> *Data collection: this section needs to use subtitle ---- allowing stating data collected at each stage for clarity.*

Thank you for this suggestion. We agree that clearly reporting timing of data collection is essential. In the present manuscript, however, we intentionally organized the section by outcome domains (functional, cognitive, psychological, and QoL measures), while explicitly reporting the timing of administration within each domain. We believe that this structure better reflects the multidimensional focus of the study and facilitates interpretation of the outcomes, while still ensuring clarity on assessment stages. For these reasons, we preferred to retain the current organization of the section.

> *Line 190: all tests were adjusted for age and education status of the participants??? Elaborate please.*

Thank you for this precious request. We clarified that raw scores obtained from neuropsychological tests were adjusted for age and education to obtain standardized scores comparable with normative data. A brief clarification was added in “Data collection” section (in bold):

> “Cognitive functioning was assessed through a comprehensive battery of neuropsychological tests. These included the Montreal Cognitive Assessment (MoCA) [39], which assessed global cognition (permission to use for research purposes was requested and training certification to administer the test was obtained). Attention, executive functions, and linguistic skills were further evaluated through the oral version of the Symbol Digit Modalities Test (SDMT) [40], the Trail Making Test (TMT-A and TMT-B) [41], the shortened version of the Stroop Colour Word test [42], the Frontal Assessment Battery [43], and the phonemic verbal fluency test [44] . All tests were adjusted for participants’ age and education **to generate standardized scores comparable to normative data available for each test**.”

> *Also, for tools used please refer to their validity and reliability (if possible).*

Thank for this comment. To avoid overloading the Methods section with psychometric details, we did not report validity and reliability indices within the manuscript. However, a reference to the published study protocol, which provides a detailed description of the psychometric proprieties of all instruments has been added (in bold):

> “[…] all participants were evaluated through a multidimensional battery of standardized scales and questionnaires, whose full description, **including their psychometric properties**, is provided in the original study protocol published elsewhere [25].”

> *Never mention how the sample size was estimated and how the study power was calculated, what effect size used for each? Possible confounding and methods to adjust them were not given?*

Thank you for these important comments. Given the pilot and feasibility-oriented nature of the study, a formal prior sample size estimation and power calculation were not performed. Instead, sample size was determined pragmatically and is consistent with methodological recommendations for pilot and feasibility studies in motor interventions, which suggest that small samples (as few as six participants per group) may be sufficient to assess feasibility and inform the design of future confirmatory trials (please see ref. 95). This aspect was explicitly acknowledged as a limitation of the study.
Regarding potential confounding factors, we acknowledge that the non-randomized design may introduce residual confounding. To mitigate this issue, participants were allocated following standardized institutional clinical procedures, and outcome analyses focused primarily on within- and between-group changes over time rather that on causal inference. Baseline clinical and demographic characteristics were also reported to allow assessment of group comparability. These aspects have been clarified and discussed in the “Limitations” section

> *Was there any method to quantify the does-response relationship, or at least recommend this future research direction.*

Thank you for this precious comment. No formal methods to quantify a dose-response relationship were included in the present study. Given the pilot and feasibility-oriented design, as well as the personalized and heterogeneous nature of the intervention, dose-response analyses may have been beyond our study purpose. However, we have explicitly acknowledged this aspect as an additional limitation and highlighted the investigation as an important direction for future research in “Study Limitations and Future Directions” section (integrations in bold):

"[…] Another limitation concerns the intervention characteristics **and the absence of formal methods to quantify dose-response relationship.** […] Moreover, this also aligns with the study's purpose of exploring intervention impact on a set of non-motor outcomes that are still insufficiently studied despite their recognized relevance. **Future studies should specifically address dose-response relationships by adopting controlled designs and systematically framing intervention intensity and exposure**. […]"

Results:

Table 1: what was group 1 and group 2??

Thank you for this precious note. To improve Tables 1-3 readability and interpretation, we have added a clarifying note specifying the composition of Group 1 and Group 2.

While within group, seems no statistical differences observed in the basic sociodemographics, and clinical characteristics, there were statistically significant differences between groups (age in PD) table 1???

Thank you for this comment. We would like to clarify that no statistically significant differences between Group 1 and Group 2 were observed in the basic sociodemographic or clinical characteristics within any of the three clinical sub-samples (stroke, PD and OA). This is reported in Table 1 and also described in the Results section addressing comparability between study arms. Here we report the information stated in the manuscript to avoid potential misunderstandings:

"Baseline socio-demographic and clinical characteristics of the study samples are presented in Table 1. For each of them, no significant between-group differences emerged, meaning that study participants were similar before the intervention."

What is PIADs subscales??? Please mention the tool in the methods section??

Thank you for this comment. We have added a brief description of the three PIADS subscales in the Methods section to clarify the dimension assessed by the instrument (integrations in bold):

"[…] At post-intervention, rehabilitation experience was evaluated with the Client-Centred Rehabilitation Questionnaire (CCRQ) [50], while the psychosocial impact of the technology implemented was assessed with the Psychosocial Impact of Assistive Device Scale (PIADS) [51], **which evaluates the perceived benefits of assistive technologies on functional ability and independence (i.e., ability subscale), motivation and ability to adapt to changes (i.e., adaptability subscale), and emotional well-being and self-confidence (i.e., self-esteem subscale).**"

Physical comfort in table 2 with marginally significant p value could be due to small sample in this group rather than a real difference? Please justify.

Thank you for this suggestion. We agree that marginally significant difference observed for physical comfort should be interpreted with caution and may by influenced by limited sample size. We have explicitly acknowledged this aspect in the Discussion, emphasizing that this finding should be considered preliminary and warrants confirmation in larger and powered studies (changes in bold):

"[…] A possible explanation of these changes may be found in patient-reported rehabilitation experience, which for those participating in technology-enhanced programs was perceived as better, especially in terms of emotional support and physical comfort provided. **However, the marginal statistical significance observed for physical comfort should be interpreted cautiously and warrants confirmation in larger trials, as it may partly reflect the limited sample size of this pilot study rather than a robust group difference. Overall,** this inference can be supported by prior literature supporting the benefits of patient-centred rehabilitation contexts for multiple rehabilitation outcomes, including perceived health and psychological well-being [81–83]."

What would be the between group differences reporting PIADs?

Thank you for this comment. In line with the analytical approach adopted for all other outcomes variables, no comparisons were performed across the three clinical sub-samples. Analyses were conducted within each sub-sample by comparing Group 1 and Group 2 when applicable. In the specific case of PIADS (differently from the data collected with CCRQ scale for rehabilitation experience), this instrument was administered only to

participants undergoing technology-enhanced rehabilitation (Group 2). Therefore, between-group comparisons were not applicable.

> *Figure 2: use whisker plot instead of the line chart with mean and errors, insert the P value in addition to whisker and plot.*
> *Figure 2: please provide label for each graph*
> *Same comments for figure 3, PD.*

Thank you for this suggestion. We acknowledge that whisker plots can be informative. However, we preferred to retain line charts displaying means and variability to ensure consistency with the data reported in the previous tables, which are presented as mean values. This choice also facilitates the interpretation of longitudinal trend over time, which was a key objective of the figure. Nevertheless, we have improved the overall quality and readability of all Figures by enhancing labels and headings, and we have added p-values when between-group comparisons were statistically significant. We believe these revisions improve clarity while also preserving consistency across figures and tables.

> *Figure 2: 4 panels including HRQoL, depression and anxiety??? Why 4???*

Thank you for this note. The four panels reflect the assessment of different but complementary outcomes. Specifically, HRQoL was evaluated using two distinct instruments: the SF-12, which provides separate composite scores for physical (PCS) and mental (MCS) health status, and the EQ-VAS. Depression and anxiety were assessed using the PHQ-4. To improve clarity, we have explicitly specified SF-12 physical and mental composite scores (PCS and MCS) in the Methods section (in bold):

> "[…] HRQoL evaluation was carried out through the Short-Form Health Survey-12 (SF-12) [45], which allowed to obtain composite scores for physical **(PCS)** and mental **(MCS)** health status, and the visual analogue scale of the Euro-QoL (EQ-VAS) […]"

> *Discussion:*
>
> *No need to describe the objective and methods in this section, please provide the answer to your research question.*

Thank you for this comment. We agree that the primary focus of the Discussion should be on interpreting the study findings by answering our research question. However, we intentionally retained a brief reference to the study objectives and design at the beginning of this section to further emphasize the pilot and feasibility-oriented nature of the work and to better contextualize the interpretation of the findings that follow. We believe that this introductory paragraph helps frame the discussion across the different clinical population and, above all, prevents overinterpretation given the exploratory scope of the study.

> *Although, conclusion for each condition was provided at the end of each, the grand conclusion for the whole study was not given at the end of the manuscript???*

Thank you for this valuable suggestion. We propose to add the following paragraph as a dedicated concluding section at the end of the manuscript to provide a comprehensive summary of our results and further emphasize the pilot and feasibility-oriented approach adopted:

> **"Overall, this pilot study provides preliminary feasibility evidence supporting the integration of personalized, technology-enhanced neuromotor rehabilitation within multidisciplinary clinical pathways across heterogeneous rehabilitation populations. Despite differences in etiology and clinical profiles, patients with stroke, PD and OA showed promising trends toward multi-domain benefits, extending beyond motor recovery to include functional autonomy, psychological well-being and HRQoL. While the exploratory nature and limited sample size warrant cautious interpretation, these findings highlight the potential added value of technology-enhanced and patient-centred rehabilitation approaches in real-world settings. Future adequately powered and controlled studies are needed to confirm these results, investigate dose-response relationships, and further clarify the mechanisms underlying multi-domain recovery."**

Reviewer #3:

As the authors addressed the given comments, I suggest acceptance of the manuscript

We sincerely thank the Reviewer for the positive evaluation and for the constructive comments, which have significantly contributed to improving the quality and clarity of the manuscript.

Reviewer #4:

General Comments

The manuscript investigates the effects of technology-enhanced neuromotor rehabilitation compared to standard care across three distinct pathologies (Stroke, Parkinson's Disease, and Osteoarthritis) on non-motor outcomes. While the topic is relevant to current trends in rehabilitation, the study presents significant methodological flaws that undermine the validity of the conclusions drawn.

Major Weaknesses:

Sample Size and Statistical Power: The sample sizes, particularly for the Parkinson's Disease (PD) subgroup (Control group $n=4$), are critically low. Conducting inferential statistics (ANOVA/t-tests) on a group of 4 participants against 8 participants yields results with extremely low statistical power and high susceptibility to Type I and II errors. The results for the PD subgroup are statistically fragile and likely unreliable.
Allocation Bias: The study uses a non-randomized, convenience sampling method where allocation was based on "resource allocation" and "routine clinical practice." This introduces severe selection bias. It is highly probable that patients assigned to the technology arm had different clinical profiles or prognoses (e.g., higher cognitive function or physical capability to handle the technology) than those assigned to standard care, despite the baseline comparison in Table 1.
Heterogeneity of Intervention: The "technology-enhanced" arm is highly heterogeneous. It combines robotics (Lokomat, Armeo-Spring) and Virtual Reality (ProKin, D-Wall) depending on the patient. Lumping these distinct modalities into a single "technology" variable obscures the specific effects of the interventions.
Control of Intensity: It is unclear if the metabolic demand and cognitive load were equated between the standard care and technology groups. If the technology group worked at a higher intensity (a common feature of robotic devices), the results may be due to intensity rather than the modality itself.

Minor Weaknesses:

Outcome Multiplicity: The study assesses a high number of dependent variables relative to the sample size. While Bonferroni corrections are mentioned, the risk of spurious significant findings remains high given the exploratory nature of the analysis.
Follow-up Attrition: There is a notable loss to follow-up (from $n=68$ enrolled to $n=52$ at 6 months). The analysis of the longitudinal data does not appear to adequately account for the characteristics of the dropouts.

We thank the Reviewer for the thorough and thoughtful evaluation of the manuscript. We acknowledge that, from a methodological perspective, the study presents several limitations, including small sample sizes, non-randomized allocation, intervention heterogeneity, and the exploratory nature of the analyses. These aspects are inherent to the pilot and feasibility-oriented design of the study and have been explicitly reported and discussed in the revised manuscript.

In response to Reviewer's comments, we have strengthened the manuscript by clarifying the feasibility scope of the investigation, carefully contextualizing the interpretation of the results, and expanding the discussion of methodological limitations, potential sources of bias, and implication for future research. Detailed responses to each specific concern raised are provided point by point, together with corresponding revisions to the manuscript.

We believe that these revisions improve the transparency and interpretability of the study, while framing the finings as preliminary evidence intended to inform the design of future adequately powered and controlled trials.

Specific Comments

Abstract

Page 1, Line 57: The term "pilot feasibility evidence" is used here, but the conclusion (Line 79) claims "widespread effectiveness." These terms are contradictory. If this is a pilot feasibility study, effectiveness cannot be firmly established.

Thank you for this important note. We agree that the wording was not fully aligned with the pilot nature of the study. To address this concern, we propose to frame the findings as preliminary and indicative of promising trends rather than established effectiveness. Here we report the revised conclusion of Abstract (changes in bold):

"Conclusion

Further full-scale trials **are warranted to confirm the longitudinal trends observed in this pilot study and to further investigate the potential multi-domain benefits** of multidisciplinary and technology-integrated rehabilitation approaches across **different** clinical populations."

Page 1, Line 62: Provide numerical data (p-values or effect sizes) for the between-group differences rather than just listing the domains.

Page 1, Line 71: Clarify if the "significant time effects" at 6 months were sustained improvements or declines compared to post-intervention.

Thank you for these suggestions. To improve the informativeness of the Abstract, we have revised the results section by adding key p-values where appropriate and clarifying that the significant time effects observed at 6-months reflect sustained improvements compared to post-intervention. Revisions were made while maintaining a concise format consistent with abstract requirements (integrations in bold):

"Results

At post-intervention, significant multi-domain intra-group improvements were observed within each sub-sample. Between-group differences were found on ADLs autonomy (stroke and PD**;** $p<.05$), executive functions (stroke; $p<.01$), anxiety and depression (OA and PD, respectively**;** $p<.05$), and well-being (stroke and OA; $p<.05$). Interaction effects were significant only on well-being variables in stroke **(**$p=.01$**)** and OA **(**$p=.02$**)**, evidencing wider short-term effects of technology-enhanced programs **compared to standard training**. At 6-month, significant time effects **indicating sustained improvements over the three timepoints** were estimated on HRQoL within each sub-sample **(**$p<.05$**)** and, additionally, on anxiety and depression in stroke **(**$p=.02$**)** and OA **(**$p<.001$**)**. Interaction effects emerged only on physical HRQoL in OA **(**$p=.02$**)**, along with significant between-group differences on HRQoL and anxiety and depression in OA **(**$p<.05$**)** and PD **(**$p=.01$**)**, respectively."

Introduction

Page 3, Line 95: The background section introduces emerging technologies but fails to adequately frame the current scope of motion analysis and wearable technology, which are the foundational mechanisms of the devices used in this study (e.g., TecnoBody). To better contextualize the "technology-enhanced" aspect, the introduction must reference recent scoping reviews that link these technologies to injury prevention and motion analysis.
Reference to add: [Souaifi M, Dhahbi W, Jebabli N, Ceylan Hİ, Boujabli M, Muntean RI, Dergaa I: Artificial intelligence in sports biomechanics: A scoping review on wearable technology, motion analysis, and injury prevention. Bioengineering 2025, 12(8):887.]

Thank you for this comment. To better contextualize the "technology-enhanced" framework, we have briefly expanded the Introduction by referring to recent literature on the role of motion analysis wearable sensor-based technologies as mechanisms underlying several rehabilitation devices used also in this study, including their contribution to functional assessment (reference added and changes in bold):

"[…] Overall, when targeting rehabilitation outcomes in these three pathologies, the adoption of multidimensional and innovative approaches is therefore essential. **Recently, advances in motion analysis and wearable sensor-based technologies have further supported technological rehabilitation by enabling objective movement assessment and personalized training planning and implementation [24]. However,** although the efficacy of technology-enhanced interventions to improve motor impairments has been supported widely […]"

Page 4, Line 169: The discussion of "individualized recovery pathways" and "rehabilitation objectives" lacks a strong theoretical basis regarding the optimization of rehabilitation risks. The manuscript should acknowledge contemporary frameworks that integrate biomechanics with rehabilitation optimization to support the "personalized approach" claimed in the study design.
Reference to add: [Dhahbi W: Advancing biomechanics: enhancing sports performance, mitigating injury risks, and optimizing athlete rehabilitation. In., vol. 7: Frontiers Media SA; 2025: 1556024.]

Thank you for this valuable suggestion. We agree that contemporary biomechanical frameworks can contribute to strengthening the theoretical basis of personalized rehabilitation pathways, particularly in relation to risk optimization and functional recovery. Accordingly, we have integrated the Introduction by briefly acknowledging the role of biomechanics-informed approaches in supporting individualized planning (integration in bold):

"[…] Overall, when targeting rehabilitation outcomes in these three pathologies, the adoption of multidimensional and innovative approaches is therefore essential. Recently, advances in motion analysis and wearable sensor-based technologies have further supported technological rehabilitation by enabling objective movement assessment and personalized training planning and implementation [24]. **In parallel, emerging biomechanics-informed rehabilitation models have increasingly contributed to optimizing individualized recovery pathways by supporting risk monitoring and functional outcome optimization.** However, although the efficacy of technology-enhanced interventions to improve motor impairments has been supported widely […]"

Page 4, Line 171: The aim states to provide "evidence on the effectiveness." Given the non-randomized design and small sample, the aim should be downgraded to "exploring preliminary trends" or "assessing feasibility."

Thank you for this precious suggestion. We have edited the aim accordingly to better clarify the pilot nature and feasibility-oriented approach of the study (changes in bold):

"[…] Following this line, the present study aimed to provide **pilot multi-domain feasibility evidence** on the impact of multidisciplinary, individualized, technology-enhanced neuromotor rehabilitation programs. **Feasibility was intended as the preliminary assessment of the applicability and potential added value of technology-integrated rehabilitation within real-world clinical settings, as well as its sensitivity in capturing changes across multiple outcome domains**. For this purpose, a preliminary longitudinal investigation was conducted […]."

Page 5, Line 186: The hypothesis that technology participants would report "significantly wider multi-domain improvements" is ambitious given the heterogeneity of the chosen technologies.

Thank you for this important observation. We agree that the original formulation of the hypotheses could appear overly ambitious considering the heterogeneity of the technological intervention adopted. Accordingly, we have revised the study hypothesis to better align it with the pilot and exploratory nature of the study, moderating the expected effects and focusing on potential short-term trends and rehabilitation experience rather than definitive effectiveness outcomes (changes in bold):

"[…] For this purpose, a preliminary longitudinal investigation was conducted […]. **In line with the pilot and exploratory nature of the study, it was hypothesized that, compared to patients undergoing standard multidisciplinary rehabilitation alone, those participating also in technology-enhanced programs would show better short-term multi-domain trends and a more positive rehabilitation experience. It was further exploratorily expected that improvements in psychological well-being and HRQoL would be at least partially maintained at follow-up**."

Methods

Page 5, Line 206: Explicitly state the exclusion criteria regarding prior use of these technologies. Prior exposure could influence the "psychosocial impact" scores due to a lack of novelty effect.

Thank you for this relevant observation. We agree that prior exposure to rehabilitation technologies could potentially influence psychosocial impact and technology experience of use outcomes. Accordingly, we have integrated the eligibility criteria section as follows (changes in bold):

"[…] In a real-world rehabilitation setting, consecutive enrolments were conducted (from September 1st 2021 to July 31th 2022) with patients meeting the following eligibility criteria: 18 years of age or older; diagnosis of stroke, PD or TKA/THA due to OA, requiring rehabilitation intervention; no severe clinical condition (i.e., chronic heart failure at class IV according to the New York Heart Association classification – NYHA-IV, ischemic heart disease at class IV according to Canadian Cardiovascular Society classification – CCS-IV, neoplastic diseases or acute respiratory disease); no cognitive impairment (screened with the Montreal Cognitive Assessment – MoCA >15.5 [26]); no

language disorders (e.g., aphasia); no severe mental health condition or psychiatric disorders (assessed according to the Diagnostic and Statistical Manual of Mental Disorders – DSM-V-TR) that can potentially compromise participation in the study and reliability of collected data**, and no prior structured exposure to the rehabilitation technologies implemented in this study**."

Page 6, Line 251: The allocation method "resulted from Institution's routine effort to optimize resource allocation" is the critical methodological flaw. Define exactly how a clinician decided a patient received technology vs. standard care. Was it based on cognitive capacity? Physical severity? This implies the groups were not comparable by prognosis.

Thank you for this methodological observation. We agree that further clarification of the allocation procedure is necessary. In the present real-world pilot study, assignment to technology-enhanced rehabilitation was not based on a single clinical characteristic (eg., cognitive profile or physical severity) but rather resulted from a multidisciplinary clinical decision-making process grounded in the integration of ICD diagnosis and ICF-based functional profiling. Specifically, allocation depended on the individual rehabilitation objectives, patient-specific functional needs, devices-specific eligibility criteria, and safety considerations, consistently with established rehabilitation technology guidelines and recommendations. We acknowledge that this real-world, non-randomized allocation may introduce potential selection bias and prognostic non-equivalence between groups. To address this concern, we have added this point as a limitation of the study (integration in bold):

"[…] As already described, patients participated in different rehabilitation activities and experienced the use of different technological devices, at different dose, based on their individual rehabilitation project and program. **Additionally, the non-randomized allocation based on routine multidisciplinary clinical decision-making may have introduced potential selection bias and prognostic non-equivalence between study groups, thus limiting causal inference and findings generalizability.** Although this contributed to obtaining greater intervention heterogeneity, the multidisciplinary and tailored approach adopted reflects the attempt to place the present study within a personalized medicine framework, whose adoption still represents one of the major challenges in the field of rehabilitation medicine [3,28,96] […]"

Page 7, Line 256: "Resulting in the same amount of therapy for all participants." Time-matched does not mean dose-matched. Provide data or a statement on how physiological or kinematic intensity was monitored to ensure equivalence.

Thank you for this important methodological remark. We agree that time equivalence does not necessarily correspond to dose equivalence. Although formal physiological or kinematic quantitative monitoring was not collected as part of the study protocol, intervention intensity and workload were routinely supervised within the clinical setting by rehabilitation professionals through functional monitoring, device-specific performance indicators, and adherence to standardized rehabilitation technology implementation guidelines. To improve clarity, we propose the following integration in methods section (in bold):

"[…] resulting in the same amount of therapy for all participants. **Also, intervention intensity and workload were routinely monitored through functional performance observation, device-generated training parameters (e.g., assistance levels, task difficulty, and progression criteria) to ensure comparable therapeutic exposure across groups** […]"

Page 7, Line 258: Combining Lokomat (gait robot), Armeo (upper limb exoskeleton), and ProKin (balance board) into one independent variable is problematic. These target different motor systems. Acknowledge this limitation in the design section.

Thank you for this relevant observation. We agree that grouping different rehabilitation technologies targeting distinct outcome domains may introduce intervention heterogeneity. The choice to consider these devices under a single technology-enhanced rehabilitation variable was driven by the study's aim to evaluate the feasibility and multidimensional impact personalized, technology-integrated rehabilitation programs within real-world clinical framework rather than device-specific effects. To address this issue, we integrated the limitation paragraph with the following change (in bold):

"Additionally, the non-randomized allocation based on routine multidisciplinary clinical decision-making **and the grouping of heterogenous technological devices under a single intervention variable** may have introduced potential selection bias and prognostic non-equivalence between study groups, thus limiting causal inference and findings generalizability."

Page 8, Line 309: Specify if the assessor for the 6-month follow-up was blinded to the group allocation.

Thank for this note. As specified in the method section, the study was conducted using an open-label design. Therefore, outcome assessors, including those involved in the 6-month follow-up evaluation, were not blinded to group allocation.

Results

Page 9, Table 1: The PD Control group has an $n=4$. Standard deviations and p-values derived from a sample of 4 are statistically unstable. The lack of significant difference at baseline ($p > 0.05$) does not prove equivalence when power is this low.

Thank you for this comment. We fully agree that, given the small sample size of the PD control subgroup, inferential statistics are statistically unstable and that non-significant baseline differences cannot be interpreted as evidence of equivalence. Baseline comparisons were included in line with common practice in pilot and feasibility studies and were not intended to demonstrate group equivalence. We have therefore clarified in the manuscript that also baseline analyses should be interpreted cautiously and that limited statistical power, particularly within PD sub-sample, is a study limitation. This aspect has been further emphasized in the Limitation section, reinforcing that findings are exploratory and hypothesis-generating rather than confirmatory (changes in bold):

> "Overall, some limitations should be acknowledged from this study. Firstly, the sample sizes of all study groups are limited**, especially in PD subsample, with consequent reduced stability of inferential estimates**. According to guidelines for pilot studies, however, a convenience sample of at least six participants per group may be sufficient for investigating recovery outcomes in pilot rehabilitation trials that are at a feasibility stage [95]."

Page 11, Table 2: The statistical comparison of "Rehabilitation Experience" between groups is questionable given the open-label nature. Patients receiving novel, expensive technology (robots) are inherently likely to report higher satisfaction due to the perceived value of the care (placebo/expectancy effects) rather than the clinical efficacy.

Thank you for this important observation. We agree that, given the open-label design, patient-reported rehabilitation experience may be influenced by expectancy and novelty effects related to the perceived value of technology-assisted care, rather than reflecting clinical efficacy. For this reason, rehabilitation experience outcomes were actually interpreted as complementary and exploratory measures to presumably support the findings observed across the other outcomes, and not as direct indicators of treatment effectiveness.

Page 12, Table 3: The notation in this table is cluttered and difficult to interpret. Ensure that the "Between-group differences" column clearly indicates which group was superior.

Thank you for this comment. We have edited Table 3 notes to improve readability and, especially, to ensure correct interpretation of between-group differences at post-intervention.

Page 14, Line 490 (PD Results): The manuscript reports significant improvements in the PD technology group ($n=8$) but not the control ($n=4$). Attributing this to the intervention is flawed; it is equally likely that the control group was simply too small to detect the natural recovery or training effect that occurred.

Thank you for this remark. We agree that, given the small size of the PD control group, the absence of significant changes cannot be interpreted as evidence of lack of improvement, nor can between-group differences be causally attributed to the intervention. As already stated in the manuscript, findings in the PD sub-sample are interpreted cautiously and framed as exploratory, with results discussed in terms of observed trends rather than definitive intervention effects. The limited statistical power of the PD control group is acknowledged as a key constraint, and the interpretation of these findings is therefore restricted to hypothesis generation rather than causal inference.

Discussion

Page 16, Line 587: The statement "patients reported significant changes in motor disability" must be contextualized by the fact that both groups improved, and no between-group difference was found for motor disability.

Thank you for this precious suggestion which helped us to better clarify that motor disability improved within both groups but without evidencing superiority. To address this aspect, we propose the following integration (in bold):

"Stroke patients reported widespread improvements after the intervention. In both study arms, patients reported significant changes in motor disability, **but no group superiority was found. Regarding levels of independence in the ADLs,** only those participating in technology-enhanced treatment showed significant improvements **at post-intervention**. […]"

Page 17, Line 620: The discussion on HRQoL improvements in Stroke lacks a critical evaluation of the "novelty effect" of technology on mood and perceived well-being, separate from physiological recovery.

Thank you for this relevant comment. We agree that improvements in HRQoL and mood following technology exposure may be partially influenced by novelty and expectancy-related effects. To address this point, we have integrated the Discussion by explicitly acknowledging the potential contribution of novelty effect on mood and perceived well-being, alongside functional recovery mechanisms (integration in bold):

"[…] The integration of technological devices not only may have provided study participants with more engaging therapy sessions and better rehabilitation experience**, with a possible contribution of novelty-related and expectancy effects on perceived well-being,** but it has also significantly contributed to improve autonomy in ADLs and cognition that, in turn, may have positively affected mood and satisfaction with it.

Page 18, Line 651: The discussion of PD results must explicitly and repeatedly acknowledge the $n=4$ control group limitation. Discussing "effectiveness" or comparing these groups as if they were balanced cohorts is misleading.

Thank you for this precious note. We fully agree that, given the small sample size, any interpretation in terms of "effectiveness" would be inappropriate and potentially misleading. Accordingly, we have revised Discussion section on PD results replacing terms implying effectiveness with more cautious language (e.g., trends, patterns, exploratory finding) where appropriate.

Page 19, Line 704: The claim that these results "corroborate" the added value of providing comprehensive recovery is too strong. The findings are correlational at best due to the lack of randomization.

Thank you for this observation. To address this point, we have revised by replacing the term "corroborate" with a more cautious wording that better reflects the exploratory nature of the evidence. The revised text now emphasized that the finings "support" or are "consistent with" prior works suggesting the added value of comprehensive and technology-enhanced recovery.

Page 20, Line 746 (OA Discussion): The analysis of motor function improvements in the OA group is superficial. It misses a critical comparison between the "focused" standard care provided and modern joint-by-joint training paradigms. This comparison is essential to understand why technology (which often engages multiple joints) might or might not offer superior outcomes compared to traditional strengthening.
Reference to add: [Dhahbi W, Materne O, Chamari K: Rethinking knee injury prevention strategies: joint-by-joint training approach paradigm versus traditional focused knee strengthening. Biology of Sport 2025, 42(4):59-65.]

Thank you for this comment. We agree that a stronger theoretical contextualization on motor improvements was needed. Accordingly, we have expanded the OA discussion by briefly contrasting focused post-arthroplasty rehabilitation with contemporary joint-by-joint training paradigms, clarifying why technology may not show superiority over modern standard care. The suggested care has been incorporate and the integration is reported below (in bold):

"[…] therefore suggesting that technology-based training was not superior in improving functional outcomes when compared to standard treatment. **This finding may reflect the characteristics of contemporary post-**

arthroplasty rehabilitation, which increasingly relies on functional, task-oriented, and multi-joint training approaches rather than isolated joint strengthening [86], potentially limiting the additional motor benefits attributable to technology-assisted training. Furthermore, this result is partially in line with prior studies that supported the efficacy of VR rehabilitation technologies but, in some cases, lacked significant comparisons with control interventions [9,87] […]"

Page 22, Line 828: The authors defend the heterogeneity as "personalized medicine." While clinically valid, in a research context without a crossover design or larger sample, this simply introduces noise. Rephrase this to acknowledge it as a confounder rather than a design strength.

Thank you for this comment. We agree that, from a research perspective, intervention heterogeneity should be acknowledged as a potential confounder rather than a design strength. Accordingly, we have revised the text to reframe heterogeneity as a limitation that may introduce variability and reduce inferential precision (changes in bold):

"[…] Although this contributed to obtaining greater intervention heterogeneity, the multidisciplinary and tailored approach adopted reflects the attempt to place the present study within a personalized medicine framework, whose adoption still represents one of the major challenges in the field of rehabilitation medicine [3,28,96]. Moreover, this also aligns with the study's purpose of exploring intervention impact on a set of non-motor outcomes that are still insufficiently studied despite their recognized relevance. **Nevertheless, it must be acknowledged that, in the present study, intervention variability may have introduced a confounding effect, sensibly limiting causal inferences**. Moving forward, future studies should specifically address dose-response relationships by adopting controlled designs and systematically framing intervention intensity and exposure. […]"

Page 22, Line 831 (Future Directions): The suggestion for future research is generic. The conclusion ignores the specific potential of machine learning and deep learning applications in biomechanical analysis, which represents the next logical step for enhancing the efficacy and personalization of the technologies tested in this pilot study.
Reference to add: [Dhahbi W, Jebabli N, Boujabli M, Souaifi M, Dergaa I, Ben Ezzdine L: MACHINE LEARNING AND DEEP LEARNING APPLICATIONS IN SPORTS BIOMECHANICAL ANALYSIS: A SYSTEMATIC SCOPING REVIEW OF PERFORMANCE ENHANCEMENT AND INJURY PREVENTION STRATEGIES. ISBS Proceedings Archive 2025, 43(1):18.]

Thank for this precious suggestion. We agree that future directions can be further improved by citing examples of tools that can prospectively enhance the efficacy and personalization of training. The implementation of AI-powered measures may be considered suitable as a future step of the present pilot evidence. Accordingly, we propose the following integration (in bold):

"[…] Moving forward, future studies should specifically dose-response relationships while adopting controlled designs and systematically framing intervention intensity and exposure. **Moreover, next research from the pilot evidence here reported may leverage the implementation of further cutting-edge tools (e.g., machine learning, artificial intelligence) to enhance training personalization and potentially enable a deeper analysis and understanding of non-motor mechanisms underlying technology-assisted recovery [99].**

Conclusions

Page 2, Line 77: The conclusion "confirming the widespread effectiveness" is not supported by the data. The study confirms feasibility and suggests potential benefits, but "confirming effectiveness" requires a powered RCT. Rephrase to reflect the pilot nature of the data.

Thank you for this precious comment. As previously reported, we have edited conclusions section to avoid confirming inferences and, rather, evidencing feasibility and warranting further investigation based on the pilot results (changes in bold):

"Further full-scale trials **are warranted to confirm the longitudinal trends observed in this pilot study and to further investigate the potential multi-domain benefits** of multidisciplinary and technology-integrated rehabilitation approaches across **different** clinical populations"
